# Dopaminergic Modulation of Effort-Related Choice Behavior as Assessed by a Progressive Ratio Chow Feeding Choice Task: Pharmacological Studies and the Role of Individual Differences

**DOI:** 10.1371/journal.pone.0047934

**Published:** 2012-10-22

**Authors:** Patrick A. Randall, Marta Pardo, Eric J. Nunes, Laura López Cruz, V. Kiran Vemuri, Alex Makriyannis, Younis Baqi, Christa E. Müller, Mercè Correa, John D. Salamone

**Affiliations:** 1 Department of Psychology, University of Connecticut, Storrs, Connecticut, United States of America; 2 Àrea de Psicobiologia, Campus de Riu Sec, Universitat Jaume I, Castelló, Spain; 3 Center for Drug Discovery, Northeastern University, Boston, Massachusetts, United States of America; 4 Pharma-Zentrum Bonn, Pharmazeutisches Institut, Pharmazeutische Chemie, Universität Bonn, Bonn, Germany; University of Chicago, United States of America

## Abstract

Mesolimbic dopamine (DA) is involved in behavioral activation and effort-related processes. Rats with impaired DA transmission reallocate their instrumental behavior away from food-reinforced tasks with high response requirements, and instead select less effortful food-seeking behaviors. In the present study, the effects of several drug treatments were assessed using a progressive ratio (PROG)/chow feeding concurrent choice task. With this task, rats can lever press on a PROG schedule reinforced by a preferred high-carbohydrate food pellet, or alternatively approach and consume the less-preferred but concurrently available laboratory chow. Rats pass through each ratio level 15 times, after which the ratio requirement is incremented by one additional response. The DA D_2_ antagonist haloperidol (0.025–0.1 mg/kg) reduced number of lever presses and highest ratio achieved but did not reduce chow intake. In contrast, the adenosine A_2A_ antagonist MSX-3 increased lever presses and highest ratio achieved, but decreased chow consumption. The cannabinoid CB1 inverse agonist and putative appetite suppressant AM251 decreased lever presses, highest ratio achieved, and chow intake; this effect was similar to that produced by pre-feeding. Furthermore, DA-related signal transduction activity (pDARPP-32(Thr34) expression) was greater in nucleus accumbens core of high responders (rats with high lever pressing output) compared to low responders. Thus, the effects of DA antagonism differed greatly from those produced by pre-feeding or reduced CB1 transmission, and it appears unlikely that haloperidol reduces PROG responding because of a general reduction in primary food motivation or the unconditioned reinforcing properties of food. Furthermore, accumbens core signal transduction activity is related to individual differences in work output.

## Introduction

Brain dopamine (DA), particularly in nucleus accumbens, plays an important role in regulating aspects of instrumental behavior [1]–[4]. Accumbens DA is involved in approach behavior, activational aspects of motivation (e.g. vigor, persistence), and enabling organisms to overcome work-related response costs to gain access to significant stimuli [1]–[13]. The increased activity induced by scheduled presentation of food pellets is accompanied by increases in accumbens DA release, and is reduced by DA antagonism and accumbens DA depletions [5], [14]. Rats with accumbens DA depletions are very sensitive to ratio requirements in operant schedules [15]–[17]. Moreover, DA antagonism or interference with accumbens DA transmission alters response allocation in tasks that measure effort-related choice behavior [1], [2], [6], [7].

Several behavioral tasks have been used to investigate the role of DA in effort-related choice. Some studies have used a T-maze barrier choice task, and reported that interference with DA transmission decreased barrier climbing to gain access to a high magnitude of reinforcement [12], [18]–[22]. T-maze and lever pressing versions of effort discounting procedures also have demonstrated that DA antagonism shifts choice behavior of rats towards low effort alternatives [23], [24]. Another task that has been used is the concurrent fixed-ratio 5 (FR5)/chow feeding procedure, in which rats can either lever press on a FR5 schedule for preferred high-carbohydrate food pellets, or approach and consume less-preferred rodent chow that is freely available in the chamber [1], [6], [25]. Under baseline conditions, rats tested with this procedure typically obtain most of their food by lever pressing while consuming very little of the chow. Systemic or intra-accumbens administration of DA antagonists and accumbens DA depletions shift response allocation such that lever pressing is decreased but chow intake is substantially increased [6], [25]–[31]. This effect is not due to drug-induced changes in food preference or consumption [6], [28]. Moreover, the effects induced by DA antagonism or depletion differ substantially from those seen following pre-feeding [6] or treatment with appetite suppressant drugs such as fenfluramine [25] or cannabinoid CB1 antagonism [30]; these appetite-related manipulations failed to increase chow intake at doses that suppress lever pressing.

In addition to accumbens DA, there is a body of research implicating adenosine in behavioral activation and effort-related processes [32]-[34]. Adenosine A_2A_ receptors are primarily localized in striatal areas, including both neostriatum and accumbens [35], [36]. Furthermore, there is a functional interaction between DA D_2_ and adenosine A_2A_ receptors [37]–[39]. Intra-accumbens injections of the adenosine A_2A_ agonist CGS 21680 decreased lever pressing on a ratio schedule [34], and also produced changes in effort-related choice behavior similar to the effects of DA antagonism [33]. In contrast, adenosine A_2A_ antagonists have been shown to increase fixed interval response rate [40]. Furthermore, several studies have shown that adenosine A_2A_ antagonists can reverse the effects of DA D_2_ antagonists on tests of effort-related choice behavior [21], [22], [31], [32], [41], [42].

The present studies employed a variant of the lever pressing/chow intake choice procedure, which utilized a progressive ratio (PROG) work requirement (see also refs. [13], [43]). Similar to the FR5/chow feeding choice task, rats tested with this PROG/chow feeding procedure have the choice of pressing the lever reinforced by presentation of the more preferred pellets vs. approaching and consuming the less preferred chow; the difference is that a PROG schedule, which gradually increases the ratio requirement, is used instead of an FR5. This variant of the choice procedure was used because the PROG schedule requires that the animal repeatedly make within-session choices between lever pressing and chow intake under conditions in which the ratio requirement was gradually incrementing. Moreover, preliminary data indicated that the PROG/chow feeding choice procedure generates much more variability in behavior between animals than the FR5 choice procedure, which could be particularly useful for studying neurochemical correlates of individual differences in behavior. To assess the effects of DA antagonism and compare these actions with other conditions, experiments were conducted to determine the effects of the DA D_2_ antagonist haloperidol, the adenosine A_2A_ antagonist MSX-3, the cannabinoid CB1 antagonist/inverse agonist and putative appetite suppressant AM251, and the reinforcer devaluation provided by pre-feeding. Furthermore, to investigate signal transduction activity that is potentially related to task performance, expression of DARPP-32 that is phosphorylated at the threonine 34 residue (pDARPP-32-(Thr34) [44] was measured immunohistochemically in 4 specific regions of interest. pDARPP-32(Thr34) immunoreactivity was used to provide a marker of DA-related signal transduction activity, because evidence indicates that DA acting through the D_1_ receptor and the G proteins G_s_/G_olf_ activates adenylate cyclase activity, thereby stimulating PKA-mediated phosphorylation of DARPP-32 at the Thr34 site [45], [46], [47], [48]. This experiment was conducted to determine if levels of pDARPP-32(Thr34) expression were higher in animals with high lever pressing output. Nucleus accumbens is implicated in effort-related processes, so both the core and shell divisions were analyzed for pDARPP-32(Thr34) activity following a PROG/Choice behavioral session. Because Schweimer and Hauber [43] demonstrated the importance of DA signaling in the anterior cingulate cortex in effort-related decision making, CG1 and CG2 divisions of the cingulate cortex also were analyzed.

It was hypothesized that haloperidol would affect PROG responding in a manner that was not dependent upon decreases in primary food motivation or appetite, and thus would decrease PROG lever pressing but leave chow intake intact. Moreover, it was hypothesized that MSX-3 would produce behavioral effects that would be opposite to those produced by haloperidol. Due to the putative appetite suppressant effects of interfering with cannabinoid CB1 receptor transmission [1], [30], [49], [50], it was expected that AM251, as well as pre-feeding, would decrease both lever pressing and chow consumption. Finally, it was hypothesized that accumbens DARPP-32 immunoreactivity would be greater in animals with high baseline levels of lever pressing (i.e., “high responders”) than in rats with low levels of lever pressing.

## Materials and Methods

### Animals

48 adult male Sprague-Dawley rats (Harlan, Indianapolis, IN, USA) were housed in a colony at 23°C with 12-h light/dark cycles (lights on at 0∶700 h). Rats weighed 300–350 g at the beginning of the study, and were initially food deprived to 85% of their free-feeding body weight for training. Rats were fed supplemental chow to maintain weight throughout the study, with water available ad libitum in the home cages. Despite food restriction, rats were allowed modest weight gain throughout the experiment. All animal protocols were approved by the University of Connecticut Institutional Animal Care and Use Committee, and followed NIH guidelines.

### Pharmacological Agents and Dose Selection

Haloperidol was obtained from Sigma-Aldrich (St. Louis, MO) and was dissolved in 0.2% tartaric acid solution. MSX-3 ((*E*)-phosphoric acid mono-[3-[8-[2-(3-methoxyphenyl)vinyl]-7-methyl-2,6-dioxo-1-prop-2-ynyl-1,2,6,7-tetrahydropurin-3-yl] propyl] ester disodium salt) was synthesized in the laboratory of Christa Müller (University of Bonn, Bonn, Germany). MSX-3 was dissolved in 0.9% saline and pH adjusted with 1.0 M NaOH to a final pH of 7.4. AM251 was synthesized in the laboratory of Alex Makriyannis (Center for Drug Discovery, Northeastern University, Boston, MA). AM251 was dissolved in dimethylsulfoxide (DMSO), Tween 80, and 0.9% saline at a ratio of 1∶1∶8. Doses were selected based on previous work [30], [40], [41].

### Behavioral Procedures

Preliminary studies were conducted to determine the optimal rate at which the schedule progressed (i.e., number of reinforcements per ratio level and by how much the ratio requirement increased with each level). It was found that by having to complete 15 ratios at each ratio level, rats generally lever pressed at higher levels before switching to chow. Behavioral sessions were conducted in operant conditioning chambers (28×23×23 cm^3^; Med Associates). Rats were initially trained to lever press on a continuous reinforcement schedule (30-min sessions; 45-mg high carbohydrate pellets, Bio-serv, Frenchtown, NJ, USA) for 1 week, and then were shifted to the PROG schedule (30-min sessions, 5 days/week) for several additional weeks. For PROG sessions, the ratio started at FR1 and was increased by one additional response every time 15 reinforcements were obtained (FR1×15, FR2×15, FR3×15,…). Additionally, this schedule included a “time-out” feature that deactivated the response lever if 2 minutes elapsed without a ratio being completed. Upon reaching stable baseline responding, chow was then introduced. Weighed amounts of laboratory chow (Laboratory Diet, 5P00 Prolab RMH 3000, Purina Mills, St. Louis, MO, USA; typically 15–20 g) were concurrently available on the floor of the chamber during the PROG sessions. At the end of the session, rats were removed from the chamber, and food intake was determined by weighing the remaining food (including spillage). Rats were trained until they attained stable levels of baseline lever pressing and chow intake, after which drug testing began. For most baseline days rats did not receive supplemental feeding, however, over weekends and after drug tests, rats received supplemental chow in the home cage. On baseline and drug treatment days, rats normally consumed all the operant pellets that were delivered during each session.

### Experimental Procedures

For experiments 1–3a and 4, the same group of animals was used (n = 32), while a different group of animals was used for experiment 3b (n = 16). For all experiments using drug manipulations (1, 2, 3b), all animals were given a single vehicle injection 1 week prior to the beginning of testing to habituate them to being injected. Experiments 1, 2, and 3b used a within-group design in which each rat received all drug or vehicle treatments (IP) in their particular experiment in a randomly varied order (one treatment per week). Baseline training sessions (i.e., non-drug) were conducted 4 days per week.

#### Experiment 1: Effects of the dopamine D_2_ antagonist haloperidol on PROG/chow feeding choice performance

To assess the effects of haloperidol, rats were trained on the PROG/chow procedure described above. On test days, animals received injections of 0.025, 0.05, 0.1 mg/kg haloperidol or vehicle, 50 minutes prior to behavioral testing.

#### Experiment 2: Effects of the adenosine A_2A_ antagonist MSX-3 on PROG/chow feeding choice performance

To assess the effects of the adenosine A_2A_ antagonist MSX-3, the same group of animals was used. The animals were first given 1 week off from any drug testing, but continued normal baseline training. On test days, animals received injections of 0.5, 1.0, 2.0 mg/kg MSX-3 or vehicle, 20 minutes prior to behavioral testing.

#### Experiment 3a/b: Effects of appetite manipulations on PROG/chow performance

*3a. Effects of pre-feeding to reduce food motivation.* To assess the effects of pre-feeding, the same group of animals was again given 1 week of additional baseline training after experiment 2. The night before testing, animals were taken off of food restriction and given ad libitum access to lab chow. On the test day, several hours before behavioral testing, animals were given ad libitum access to Bio-serv pellets in the home cage. Performance on the test day was then compared to performance on the previous baseline day.

*3b: Effects of the cannabinoid CB1 inverse agonist and putative appetite suppressant AM251.* For assessment of the effect of AM251, a new group of animals (n = 16) were trained on the PROG/chow feeding choice procedure described above. On test days, animals received injections of 2.0, 4.0, 8.0 mg/kg AM251 or vehicle, 30 minutes prior to testing, once per week, in a randomly varied order.

#### Experiment 4: Effects of PROG/chow responding on pDARPP32-THR34 expression: high vs. low responders

Following the conclusion of experiment 3, animals (n = 32) were given 1 week to re-stabilize their baselines. During the following week, 90 minutes after a baseline training session, animals were sacrificed and perfused to obtain tissue for pDARPP32-(Thr34) immunohistochemical analysis. For statistical analysis, these animals were divided into two groups (high performers and low performers, determined by a median split of lever pressing on the day of perfusion).

### pDARPP32(Thr34) visualization and quantification

Free floating coronal sections (50 µm) were serially cut using a cryostat (Weymouth, MA, USA) and rinsed in 0.01 M PBS (pH 7.4). pDARPP32-(Thr34) immunohistochemistry was conducted according to the methods described previously by Segovia et al. (2012). The primary anti-pDARPP32-(Thr34) antibody was used at a concentration of 1∶1000 (Santa Cruz Biotechnology, USA) for 48 h incubation, and the secondary antibody was anti-rabbit HRP conjugate, envision plus (DAKO, Carpinteria, CA, USA). The immunohistochemical reaction was developed using diaminobenzidine (DAB) as the chromagen. The mounted and cover-slipped sections were examined and photographed using a Nikon Eclipse E600 (Melville, NY, USA) microscope equipped with an Insight Spot digital camera (Diagnostic Instruments, Inc). Images of the regions of interest were magnified at 20× and captured digitally using SPOT software. Cells that were positively labeled for pDARPP32-(Thr 34) were quantified with ImageJ software (v. 1.42, National Institutes of Health sponsored image analysis program) and a macro written to automate particle counting within regions of interest (1000×1000 µm). For each animal, cell counts were obtained bilaterally from at least three sections, and counts were averaged across sides and sections. All cell counting was done by someone who was blind to the experimental conditions.

### Statistical Analysis

For the behavioral pharmacology experiments, number of lever presses, maximum ratio achieved, active lever time (in seconds) and chow intake (grams) were analyzed using repeated measures analysis of variance (ANOVA). Non-orthogonal planned comparisons using the ANOVA error term [51] were used to compare each treatment with the vehicle control. In addition, to provide another statistical measure of the reciprocal relationship between lever pressing and chow intake in each experiment, correlations were performed between number of lever presses and chow intake data collapsed across all conditions within the experiment. This measure has been used in previous studies [25], [27], and is potentially influenced by both between subject and treatment-related variability in lever pressing and chow intake. Thus, in order to control for the effect of between-subject variability on this measure, partial correlations that controlled for the vehicle level of lever pressing also were performed. For experiment 4, pDARPP32-(Thr34) cell counts were analyzed for differences in expression between high and low responders (after a median split of the lever pressing data) for each of 4 regions of interest, and t-tests were performed to determine significant differences.

## Results

### Experiment 1: Effects of the DA D_2_ antagonist haloperidol

**Figure 1:**
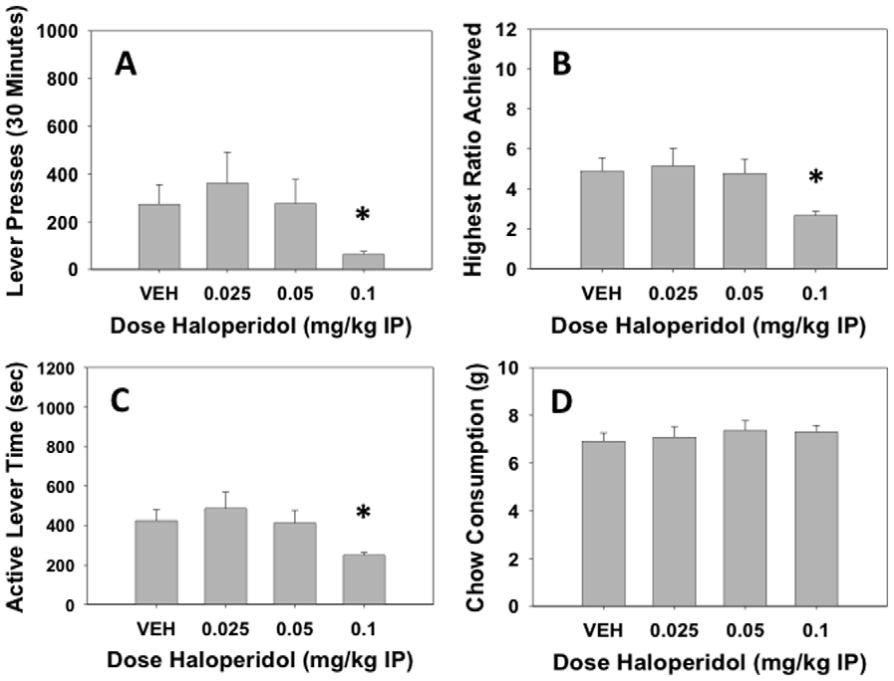
Effects of the dopamine D_2_ antagonist haloperidol on PROG/chow performance. On measures of lever pressing, mean (±SEM) total lever presses (A), maximum ratio achieved (B), and active lever time (measured in seconds, C), haloperidol significantly decreased at the highest dose of 0.1 mg/kg. Chow consumption (mean±SEM, in grams) during test sessions was unaffected by any dose tested (D). (* p<0.05, different from vehicle)

Haloperidol significantly decreased the number of lever presses (F(3,93) = 4.598, p<0.01, see figure 1A). Planned comparisons revealed that there was a significant difference between 0.1 mg/kg haloperidol and vehicle (p<0.05). Haloperidol also significantly decreased maximum ratio achieved (F(3,93) = 8.661, p<0.01, figure 1B), and the amount of time the lever remained active (F(3,93) = 6.723, p<0.01, figure 1C); for both measures, planned comparisons showed a significant difference between vehicle and 0.1 mg/kg haloperidol (p<0.05). Haloperidol produced no significant effects on chow consumption in the dose range tested (figure 1D). However, there was a tendency for animals that had high vehicle rates of responding, and correspondingly low vehicle levels of chow intake, to show increases in chow intake with haloperidol; this was marked by a significant correlation between vehicle number of lever presses and the difference in chow consumption between vehicle and the highest dose of haloperidol (r = 0.69, df = 30, p<0.05). Collapsed across all conditions, there was a significant negative correlation between number of lever presses and chow consumption (r = −0.765, df = 126, p<0.001), which demonstrated the overall inverse relationship between lever pressing and chow intake. Moreover, the partial correlation between lever pressing and chow intake that controlled for between-subject variability also was statistically significant (−0.557, df = 125, p<0.001), indicating that the inverse relation between lever pressing and chow intake was evident across treatments even if one controlled for the between-subject variability in lever pressing.

### Experiment 2: Effects of the adenosine A_2A_ antagonist MSX-3

**Figure 2:**
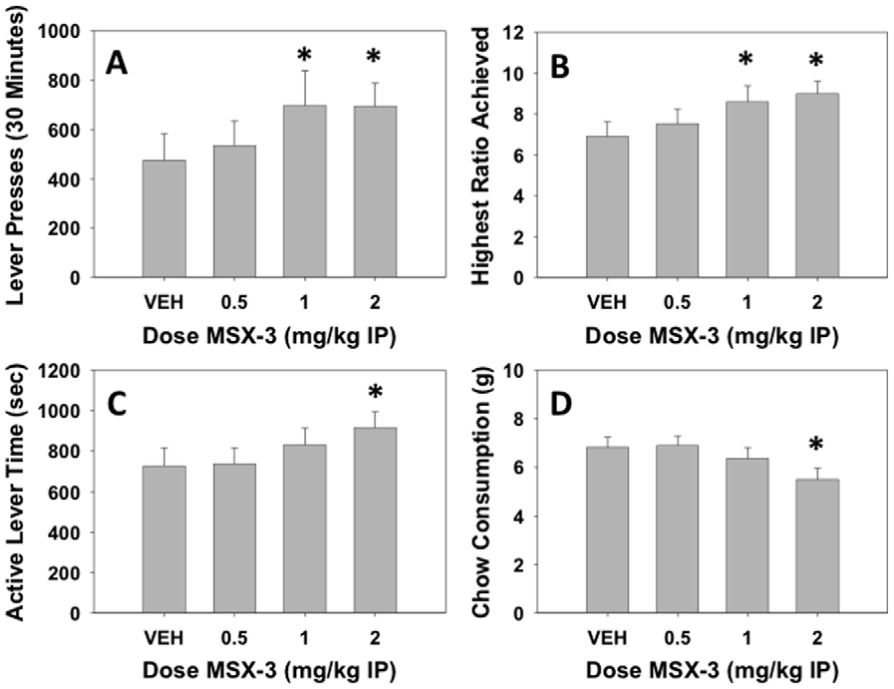
Effects of the adenosine A_2A_ antagonist MSX-3 on PROG/chow performance. On measures of lever pressing, mean (±SEM) total lever presses (A) and maximum ratio achieved (B) were both significantly increased at doses of 1.0 and 2.0 mg/kg while active lever time (measured in seconds, C) was only increased at a dose of 2.0 mg/kg. Chow consumption (mean±SEM, in grams) during test sessions was significantly decreased at the dose of 2.0 mg/kg (D). (* p<0.05, different from vehicle)

MSX-3 significantly increased number of lever presses (F(3,93) = 4.120, p<0.01, figure 2A), and planned comparisons showed that both 1.0 and 2.0 mg/kg doses of MSX-3 increased number of lever presses compared to vehicle (p<0.05). There also was a significant increase in maximum ratio achieved (F(3,93) = 8.206, p<0.01, see figure 2B), with the 1.0 and 2.0 mg/kg doses of MSX-3 differing significantly from vehicle (p<0.05). Furthermore, MSX-3 increased active lever time (F(3,93) = 3.784, p<0.05, figure 2C), with the 2.0 mg/kg does of MSX-3 being significantly affected (p<0.05). MSX-3 decreased chow intake (F(3,93) = 8.017, p<0.01; figure 2D), with a significant effect at 2.0 mg/kg MSX-3 compared to vehicle (p<0.05). As with experiment 1, there was a significant negative correlation between lever presses and chow intake when the data were collapsed across all conditions (r = −0.781, df = 126, p<0.001). In addition, the partial correlation between lever pressing and chow intake that controlled for between-subject variability also was statistically significant (−0.597, df = 125, p<0.001). Thus, as with the haloperidol experiment, the inverse relation between lever pressing and chow intake was evident across drug treatments even if one controlled for the between-subject variability in lever pressing.

### Experiment 3: Effects of appetite-related manipulations on PROG/chow performance: effects of pre-feeding and the putative appetite suppressant AM251

**Figure 3:**
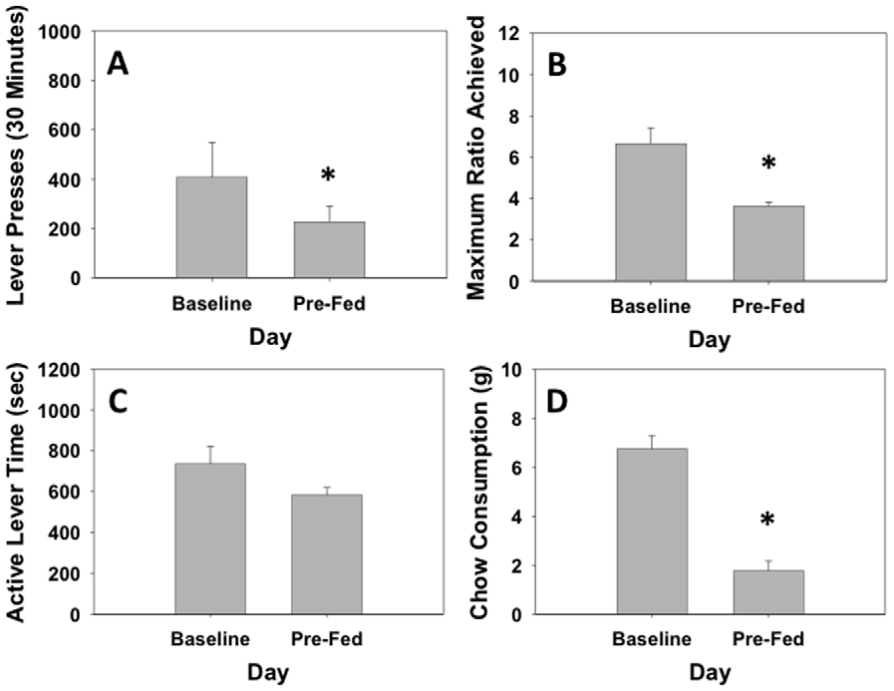
Effects of prefeeding on PROG/chow performance. On measures of lever pressing, mean (+SEM) total lever presses (A), maximum ratio achieved (B), and active lever time (measured in seconds, C) were all significantly decreased with prefeeding. Chow consumption (mean+SEM, in grams) during test sessions was significantly decreased by prefeeding animals prior to the test session (D). (* p<0.05, different from vehicle)

**Figure 4:**
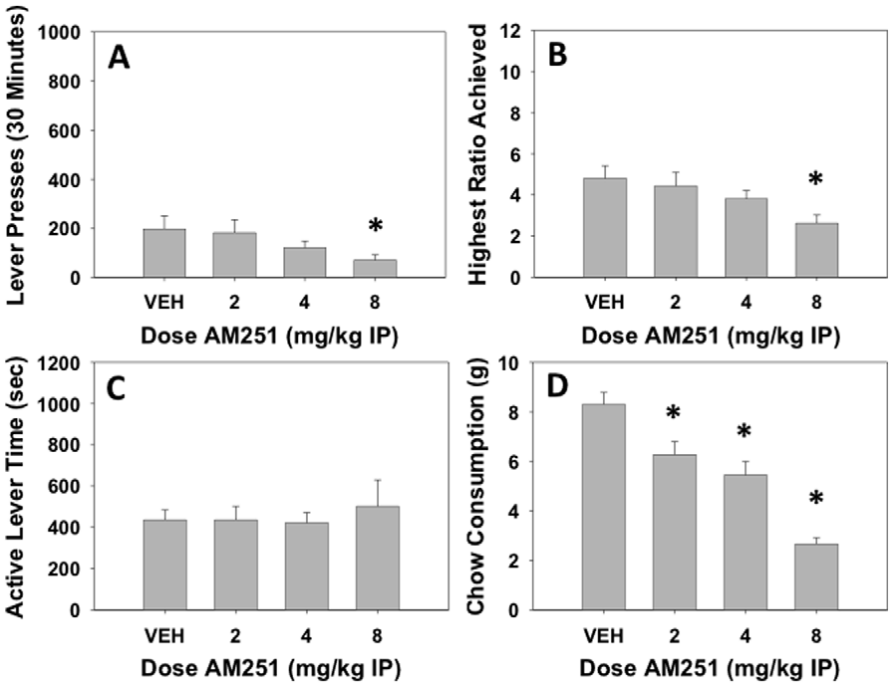
Effects of the cannabinoid CB1 inverse agonist AM251 on PROG/chow performance. On measures of lever pressing, mean (±SEM) total lever presses (A) and maximum ratio achieved (B) were significantly decreased at 8.0 mg/kg while active lever time (measured in seconds, C) was unaffected at any dose tested. Chow consumption (mean ±SEM, in grams) during test sessions was significantly decreased at 2.0, 4.0 and 8.0 mg/kg doses (D). (* p<0.05, different from vehicle)

Experiment 3a studied the effects of pre-feeding on PROG/chow intake choice performance (figures 3A–D). Compared to the previous baseline day, pre-feeding the animals prior to the session produced marked decreases in number of lever presses (t = 2.96, df = 31, p<0.05), and maximum ratio achieved (t = 3.94, df = 31, p<0.05), but no significant effect on active lever time. Pre-feeding significantly decreased chow intake (t = 13.69, df = 31, p<0.01) compared to previous day baseline performance. There was no significant overall correlation between number of lever presses and chow consumption (r = 0.12, df = 62, n.s.), and no significant partial correlation between lever pressing and chow intake when we controlled for the between-subject variability in lever pressing (r = 0.156, df = 61, n.s.). Experiment 3b studied the effects of the cannabinoid CB1 inverse agonist AM251. AM251 decreased the number of lever presses (F(3,45) = 3.891, p<0.05, figure 4A), and the maximum ratio achieved (F(3,45) = 5.811, p<0.05, see figure 4B). Planned comparisons showed that with both measures, only the highest dose of 8.0 mg/kg AM251 significantly differed from vehicle (p<0.05). AM251 did not produce any significant changes in active lever time (figure 4C), but it significantly decrease chow intake (F(3,45) = 45.634, p<0.01, figure 4D), with all doses differing from vehicle (p<0.05). There was no significant overall correlation between number of lever presses and chow consumption (r = −0.05, df = 62, n.s.), and no significant partial correlation between lever pressing and chow intake when we controlled for the between-subject variability in lever pressing (r = 0.234, df = 61, n.s.).

### Experiment 4: pDARPP-32(Thr34) Immunohistochemistry in high and low responders

**Figure 5:**
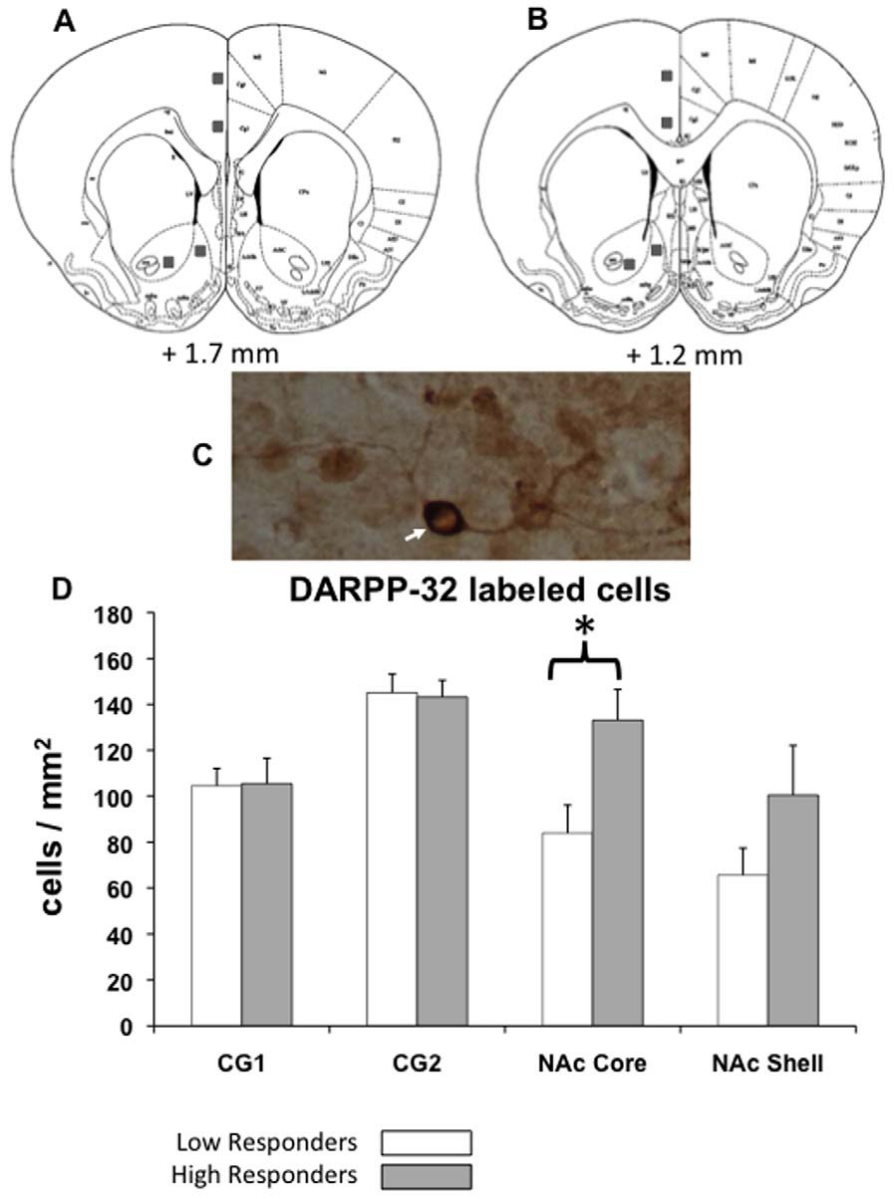
pDARPP-32(Thr34) immunocytochemistry. (A and B) Atlas plates (modified from Paxinos and Watson [68] with regions of interest denoted by squares. (C) High magnification photomicrograph of pDARPP-32(Thr34) immunoreactive cells at 40× magnification. Several pDARPP-32(Thr34) positive cells are shown, including a darkly staining cell, with clear soma and dendritic processes (arrow) (D) Mean (±SEM) number of pDARPP-32(Thr34) positive cells counted in each region of interest in high performers and low performers. There were significantly more pDARPP-32(Thr34) positive cells counted in the nucleus accumbens core of high performers compared to low performers. (* p<0.05)

**Figure 6:**
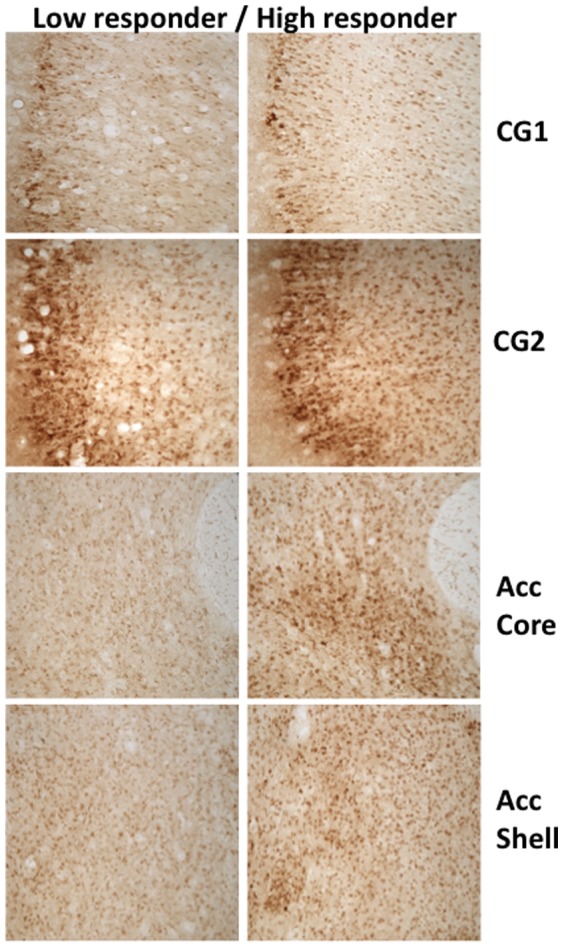
Photomicrographs of pDARPP-32(Thr34) staining in each region of interest, showing representative low performer (left column) and high performer (right column). All images were taken at 10× magnification.

Performance on the PROG/chow feeding choice task was highly variable; some rats lever pressed fewer than 100 times and had high levels of chow intake, while others lever pressed more than 1000 times and consumed small amounts of chow. This variability was seen across all the experiments described above, and in some cases was related to the drug effects seen. For example, the effects of haloperidol were more marked in rats with higher control levels of lever pressing. When a median split was done, and high and low lever pressing was used as a factor in a 2×4 factorial ANOVA, there was an overall effect of dose (F[3,90] = 5.071, p<0.05) and importantly, a dose by group interaction (F[3,90] = 4.189, p<0.05). Although the repeated measures ANOVA demonstrated that both low and high responders showed significant decreases in number of lever presses (low responders: F(3,45) = 2.790, p<0.05; high responders: F(3,45) = 4.638, p<0.05), analysis of effect sizes showed that the suppressive effect of haloperidol on number of lever presses was greater in high responders (eta^2^ = 0.236) than low responders (eta^2^ = 0.157). Similar analyses revealed differences between high and low responders in the AM251 experiment, with only the high responders showing a significant drug effect on number of lever presses. Because of this large variability, the final experiment investigated potential neurochemical differences between high and low responders, using pDARPP-32(Thr34) as a marker of signal transduction activity. To analyze the pDARPP-32(Thr34) expression data, a median split based upon behavioral performance during the final test day was performed, yielding two groups: high responders (n = 16, mean = 812.44, SEM = 201.68, range = 205–2852) and low responders (n = 16, mean = 116.31, SEM = 12.81, range = 54–190). Four regions of interest were selected for analysis: cingulate cortex CG1/CG2 and accumbens core/shell (Figures 5–6). There were no differences in pDARPP-32(Thr34) expression between high and low responders in CG1 (t = −0.066, df = 28, n.s.) or CG2 (t = 0.172, df = 25, n.s.). Nucleus accumbens shell showed no significant differences in pDARPP-32(Thr34) expression between high and low responders (t = 1.415, df = 30, n.s.), but nucleus accumbens core showed a significant difference in pDARPP-32(Thr34) expression between high and low responders (t = 2.703, df = 29, p<0.05, figures 5–6).

## Discussion

The present studies investigated the effects of several manipulations using a concurrent PROG/chow feeding task. Experiment 1 demonstrated that the DA D_2_ antagonist haloperidol decreased number of lever presses, maximum ratio achieved, and active lever time (i.e., the time the PROG schedule was active). These findings are consistent with previous studies showing the ability of DA antagonists or accumbens DA depletions to reduce food-reinforced lever pressing in animals responding on the concurrent FR5/choice task [6], [25], [30], as well as conventional operant schedules, including various versions of the progressive ratio schedule [52], [53]. Moreover, they are consistent with recent research showing that the increased DA transmission resulting from DA transporter knockdown enhanced progressive ratio performance, including conditions in which a PROG schedule reinforced by sucrose was available in the home cage and non-deprived animals also had their homecage food available [13]. Despite producing clear reductions in measures of operant responding, haloperidol did not decrease chow intake, which indicates that primary food motivation was intact in haloperidol-treated rats. Moreover, previous studies have shown that 0.1 mg/kg haloperidol does not change preference for these specific high carbohydrate food pellets relative to chow, or reduce total intake of either food type in free-feeding tests [6], [54]. In fact, there was a slight tendency for some rats to show increased chow intake after haloperidol treatment, which was marked by the significant correlation between vehicle lever pressing and the difference in chow intake between vehicle and the highest dose of haloperidol. In other words, animals that were high lever press responders under the vehicle condition, and therefore had correspondingly low levels of chow intake, showed greater increases in chow consumption on haloperidol than low responders did. In fact, the 4 rats with the highest level of lever pressing showed very substantial increases in chow intake after haloperidol injection (i.e., increases of 3–6 grams relative to vehicle). Nevertheless, unlike the previous experiments using the FR5/chow choice task [6], [25], [30], haloperidol did not produce an overall significant increase in chow intake. One possible explanation for this pattern is the different levels of chow intake with the two procedures. With the FR5/chow choice procedure, baseline or control levels of lever pressing are relatively high, while chow intake is relatively low (i.e., 1–2 grams), making it possible to observe an increase in chow intake with administration of a DA antagonist. In contrast, baseline or control levels of chow intake are much higher with the PROG/chow choice procedure (i.e., 7–8 grams), and are near ceiling levels of chow intake for a 30 minute period without water being available. For example, Randall et al. [50] demonstrated that food-restricted rats in a free feeding study consume approximately 8 grams of chow in a 30-minute period. Thus, with the PROG/chow choice procedure, it is difficult to observe drug-induced increases in chow intake in animals that are already eating chow at maximal or near maximal levels. Nevertheless, compared to the FR5/chow choice procedure, the PROG/chow choice task is better suited for observing large reductions in chow intake due to appetite-related manipulations such as pre-feeding or appetite suppressant drugs (see discussion below).

The adenosine A_2A_ antagonist MSX-3 produced effects that were opposite to those of haloperidol; MSX-3 increased number of lever presses and maximum ratio achieved, and also increased the amount of time that animals kept the lever active during the session. This is consistent with previous work showing that adenosine A_2A_ antagonists have stimulant-like properties. For example, the adenosine A_2A_ antagonists MSX-3 and istradefylline increased lever pressing on a fixed interval 4-minute schedule, which generates a relatively low baseline rate of responding [40]. In addition, the PROG/chow feeding choice procedure allowed for parallel assessment of food intake, and MSX-3 decreased chow consumption at the highest dose. Interestingly, although MSX-3 and haloperidol produced opposite effects on measures of PROG lever pressing and chow intake, in both experiments, the reciprocal relation between lever pressing and chow intake was preserved, as indicated by the high negative correlations between lever pressing and chow intake across all treatments (−0.76 and −0.78). Significant negative partial correlations also were observed in the haloperidol and MSX-3 experiments when partial correlations were used to control for between-subject variability in lever pressing. This inverse correlation between lever pressing and chow intake has been reported in previous experiments studying the effects of DA antagonists or depletions on FR5/chow feeding choice performance [25], [27], [30].

Experiment 3 was conducted to determine the effect of appetite-related manipulations on PROG/chow feeding choice performance, in order to provide a contrast with the effects of haloperidol. Two different appetite manipulations were employed: pre-feeding, and administration of a cannabinoid CB1 receptor antagonist/inverse agonist. Pre-feeding animals prior to their test session, which was used to reduce food motivation and thereby devalue the food reinforcement [15], [54], produced marked decreases in number of lever presses and highest ratio achieved. But, unlike the effects of haloperidol, pre-feeding also substantially reduced chow consumption. In experiment 3b, the CB1 receptor antagonist/inverse agonist AM251 was assessed. CB1 antagonists/inverse agonists are putative appetite suppressant drugs that have been shown to decrease food intake in animals [30], [49], [50], [55] and humans [56]. Moreover, stimulation of CB1 receptors in nucleus accumbens shell increased hedonic taste reactivity for intraoral sucrose [57]. On the PROG/chow feeding choice task, AM251 decreased number of lever presses, maximum ratio achieved, and chow consumption. Thus, the pattern of effects on lever pressing and chow intake produced by pre-feeding and AM251 differed markedly from those produced by haloperidol. Moreover, while there was a high inverse correlation between lever pressing and chow intake in the haloperidol experiment, there were no significant correlations or partial correlations between these measures in the pre-feeding and AM251 experiments. This analysis shows that the inverse relation between lever pressing and chow intake, which is evident under baseline conditions and also throughout the haloperidol experiment, is not shown when primary food motivation is reduced by pre-feeding or drugs, because appetite-suppressant manipulations decrease both food reinforced lever pressing and chow consumption [25], [30]. There are a number of factors that can influence progressive ratio performance [58], and rather than yielding a simple measure of “reward”, progressive ratio breakpoints represent the outcome of a cost/benefit analysis related partially to characteristics of the reinforcer itself, but also the work-related response costs and time constraints imposed by the ratio schedule [2]. Indeed, previous work has demonstrated that progressive ratio break points are sensitive to work-related factors such as the height of the lever [59]. Taken together, experiments 1–3 demonstrate that it is exceedingly unlikely that haloperidol is decreasing PROG lever pressing because of a reduction in primary food motivation, appetite, or the unconditioned reinforcing properties of food. Clearly, in the absence of parallel measures of food intake, or taste reactivity [57], [60], progressive ratio break points should not be used as markers of food “reward”, or “hedonic” reactivity to food [61]. Rather, they provide a measure of how much work the organism will do in order to gain access to a reinforcing stimulus [2], [61], [62].

An important aspect of the PROG/choice procedure is that performance is characterized by substantial individual variability. While some rats lever pressed relatively little and had high levels of chow intake, others lever pressed much more and ate relatively little chow. Experiment 4 employed pDARPP-32-(Thr34) immunohistochemistry to determine if there were neurochemical differences between high responders and low responders. The entire group of animals was divided in two by a median split based upon numbers of lever presses, and pDARPP-32(Thr34) expression was determined. High responders did not differ from low responders in terms of pDARPP-32(Thr34) expression in CG1 or CG2 regions of anterior cingulate cortex. In the accumbens shell, there was a slight tendency for high responders to show increased pDARPP-32 expression, but this was not statistically significant. However, high responders did show significantly greater pDARPP-32(Thr34) expression in accumbens core than low responders. pDARPP-32(Thr34) immunoreactivity was used to provide a marker of signal transduction activity, and evidence indicates that DA acting through the D_1_ receptor and related G proteins (G_s_/G_olf_) activates adenylate cyclase activity, thereby stimulating PKA-mediated phosphorylation of DARPP-32 at the Thr34 site [45]–[48]. DARPP-32 expression has been used to study drug effects [47], [48], and a few studies have focused on changes in DARPP-32 immunoreactivity associated with behavioral manipulations. Danielli et al. [63] demonstrated that pDARPP-32(Thr34) showed increased expression in nucleus accumbens shell after the first exposure to a novel food. Recently, Segovia et al. [44] reported that pDARPP-32(Thr34) expression in accumbens shell and core was increased in animals undergoing FR5 operant training. Although several neurochemical factors can influence pDARPP-32(Thr34) production [48], it is reasonable to suggest that the higher level of pDARPP-32(Thr34) expression in high responders relative to low responders could reflect greater DA release in the animals working harder on the lever pressing component of the task [44], [64]. If so, this could indicate that individual differences in work output are related to individual differences in DA transmission in striatal areas, as recently shown in a human imaging study [65]. Furthermore, these observations of individual variability in exertion of physical effort are consistent with recent studies of individual variability in cognitive effort across subjects (i.e., “workers” vs. “slackers”) [66].

## Conclusions

In summary, the DA antagonist haloperidol reduced the number of lever presses and highest ratio achieved but did not suppress chow intake. In contrast, the adenosine A_2A_ antagonist MSX-3 increased lever presses and highest ratio achieved, but decreased chow consumption. Pre-feeding and administration of the cannabinoid CB1 antagonist/inverse agonist AM251 decreased lever presses, highest ratio achieved, and chow intake. Including the option of having chow concurrently available allows one to conclude that the effects of DA antagonism differed greatly from those produced by pre-feeding or decreases in CB1 transmission, despite the fact that all three manipulations decreased lever pressing. Thus, haloperidol is not reducing PROG responding because of a general reduction in primary food motivation or the valuation of food reinforcement. Instead, the present data are consistent with the hypothesis that haloperidol left aspects of food motivation intact, but reduced the tendency to work for food reinforcement. Furthermore, DA-related signal transduction activity (pDARPP-32(Thr34) expression) was greater in high responders (i.e., rats with high lever pressing output) compared to low responders, indicating that accumbens core signal transduction activity is related to individual differences in work output. Future studies should compare the effects of DA D_1_ and D_2_ antagonists, and should determine if adenosine A_2A_ antagonism is capable of reversing the effects of DA antagonism. Studies comparing cannabinoid CB1 inverse agonists with neutral antagonists (e.g. 50, 30) would be useful for further exploration of the role of CB1 receptor signaling in performance on this procedure. Finally, additional neurochemical measures should be investigated for their possible relation to lever pressing output on this task, including microdialysis studies of DA release [64], and other markers of signal transduction activity (e.g. c-Fos, pDARPP-32(Thr75)) in different striatal cell types (e.g. encephalin or substance P positive neurons; [44]). These studies are important for understanding activational aspects of motivation, and may contribute to our understanding of the neural basis of effort-related motivational impairments (e.g. anergia, psychomotor retardation, fatigue) in depression and other disorders [1], [2], [67].
